# Astrocyte Elevated Gene-1 Cys75 *S*-Palmitoylation by ZDHHC6 Regulates Its Biological Activity

**DOI:** 10.1021/acs.biochem.2c00583

**Published:** 2022-12-22

**Authors:** Garrison Komaniecki, Maria Del Carmen Camarena, Eric Gelsleichter, Rachel Mendoza, Mark Subler, Jolene J. Windle, Mikhail G. Dozmorov, Zhao Lai, Devanand Sarkar, Hening Lin

**Affiliations:** † Department of Chemistry and Chemical Biology, Cornell University, Ithaca, New York 14853, United States; ‡ C. Kenneth and Dianne Wright Center for Clinical and Translational Research, Virginia Commonwealth University, Richmond, Virginia 23298, United States; § Department of Human and Molecular Genetics, Virginia Commonwealth University, Richmond, Virginia 23298, United States; ∥ Massey Cancer Center, Virginia Commonwealth University, Richmond, Virginia 23298, United States; ⊥ VCU Institute of Molecular Medicine (VIMM), Virginia Commonwealth University, Richmond, Virginia 23298, United States; # Department of Biostatistics, Virginia Commonwealth University, Richmond, Virginia 23298, United States; ∇ Department of Pathology, Virginia Commonwealth University, Richmond, Virginia 23298, United States; ○ Greehy Children’s Cancer Research Institute, University of Texas Health Science Center San Antonio, San Antonio, Texas 78229, United States; ◆ Howard Hughes Medical Institute, Department of Chemistry and Chemical Biology, Cornell University, Ithaca, New York 14853, United States

## Abstract

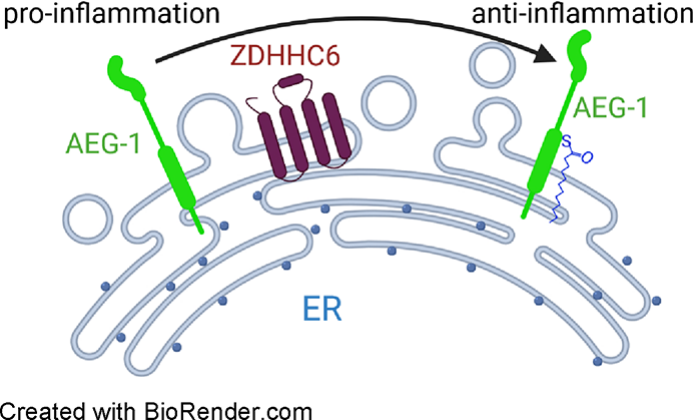

Nonalcoholic fatty liver disease is a major risk factor for hepatocellular carcinoma (HCC). Astrocyte elevated gene-1/Metadherin (AEG-1/MTDH) augments lipid accumulation (steatosis), inflammation, and tumorigenesis, thereby promoting the whole spectrum of this disease process. Targeting AEG-1 is a potential interventional strategy for nonalcoholic steatohepatitis (NASH) and HCC. Thus, proper understanding of the regulation of this molecule is essential. We found that AEG-1 is palmitoylated at residue cysteine 75 (Cys75). Mutation of Cys75 to serine (Ser) completely abolished AEG-1 palmitoylation. We identified ZDHHC6 as a palmitoyltransferase catalyzing the process in HEK293T cells. To obtain insight into how palmitoylation regulates AEG-1 function, we generated knock-in mice by CRISPR/Cas9 in which Cys75 of AEG-1 was mutated to Ser (AEG-1-C75S). No developmental or anatomical abnormality was observed between AEG-1-wild type (AEG-1-WT) and AEG-1-C75S littermates. However, global gene expression analysis by RNA-sequencing unraveled that signaling pathways and upstream regulators, which contribute to cell proliferation, motility, inflammation, angiogenesis, and lipid accumulation, were activated in AEG-1-C75S hepatocytes compared to AEG-1-WT. These findings suggest that AEG-1-C75S functions as dominant positive and that palmitoylation restricts oncogenic and NASH-promoting functions of AEG-1. We thus identify a previously unknown regulatory mechanism of AEG-1, which might help design new therapeutic strategies for NASH and HCC.

## Introduction

Nonalcoholic fatty liver disease (NAFLD), which includes nonalcoholic steatohepatitis (NASH), is a major health problem. NAFLD is characterized by initial accumulation of triglyceride in hepatocytes (hepatic steatosis) with subsequent chronic inflammation leading to NASH.^1,2^ NAFLD is the most common cause of chronic liver disease in the Western world, leading to cirrhosis and hepatocellular carcinoma (HCC).^3,4^ Some molecular players regulating NAFLD have been identified, leading to multiple strategies in clinical trials.^2^ However, the optimal therapy is yet to be developed, motivating the pursuit of clearer understandings of the molecular mechanism of NAFLD to yield effective therapeutic strategies.

Our previous studies have identified astrocyte-elevated gene-1 (AEG-1, also called metadherin or MTDH, lysine-rich CEACAM1 coisolated protein or LYRIC) as a key regulator of NASH and HCC. AEG-1 contains a single transmembrane domain near the N-terminus, which localizes it to the ER membrane, with the majority of the protein (C-terminal to the transmembrane domain) in the cytosol.^5^ AEG-1 functions as an oncogene by protein–protein and protein–RNA interaction in diverse cancers, including HCC.^6−17^ AEG-1 also promotes NASH.^18^ Hepatocyte-specific AEG-1 transgenic mice (Alb/AEG-1) develop spontaneous NASH, and hepatocyte-specific conditional AEG-1 knockout mice (AEG-1^ΔHEP^) show profound resistance to high fat diet (HFD)-induced NASH.^18^ AEG-1 levels are markedly high in biopsy samples of human NASH livers compared to normal livers.^18^ BL6/129SV mice, fed with HFD along with sugared water for 52 weeks, develop NASH, cirrhosis, and HCC with features similar to human disease (the DIAMOND mice).^19^ Marked AEG-1 overexpression was detected in steatotic livers as well as in the tumors of DIAMOND mice compared to chow-fed normal liver.^18^ Given the important regulatory roles of AEG-1 in NASH and HCC, we reasoned that it would be important to understand how AEG-1 itself is regulated because such understanding may reveal new strategies to target AEG-1 to treat NASH and HCC.

Protein *S*-acylation results from enzymatic addition of long-chain fatty acyl groups, typically C16 palmitate (hence also called S-palmitoylation), onto intracellular cysteine residues of soluble and transmembrane proteins via a labile thioester linkage.^20^ It has been reported to occur on over 2000 mammalian proteins. Addition of palmitoyl increases hydrophobicity of proteins and can impact protein structure, assembly, maturation, trafficking, and function. Palmitoylation is emerging as an important ubiquitous mechanism to control protein function and consequently physiological processes.^21^*S*-Palmitoylation is catalyzed by 23 members of the zinc finger DHHC (ZDHHC) family of acyltransferases (DHHC stands for a conserved Asp-His-His-Cys motif), which typically utilizes coenzyme A [CoA]-palmitate, and deacylation is catalyzed by a number of acyl thioesterases of the serine hydrolase family.^21^ AEG-1 is reported as a potential palmitoylated protein in many proteomic studies in SwissPalm (https://swisspalm.org/proteins/Q86UE4). However, this has not been biochemically validated. Here, we biochemically validated that AEG-1 is indeed palmitoylated. We further identified that palmitoylation occurs on Cys75 of AEG-1, ZDHHC6 is a major palmitoyltransferase for AEG-1, and palmitoylation might negatively regulate AEG-1 function.

## Materials and Methods

### Reagents

Anti-FLAG affinity gel (A2220) and anti-FLAG antibody conjugated with horseradish peroxidase (A8592, 1:10000) and HA antibody (SAB4300603, 1:1000) were purchased from Sigma-Aldrich. Antibodies against β-actin (sc-4777) were purchased from Santa Cruz Biotechnology. DHHC3 antibody (ab31837, 1:1000) and DHHC7 antibody (ab138210, 1:1000) were purchased from Abcam. Protease inhibitor cocktail was purchased from Sigma-Aldrich. Streptavidin agarose beads, ECL plus western blotting detection reagent, and universal nuclease for cell lysis were purchased from Thermo Scientific Pierce. Polyethyleneimine (PEI) was purchased from Polysciences (24765).

### Cloning and Mutagenesis

Full length AEG-1 (RefSeq: NC_000008.11) was cloned into pcDNA3.1 (C-terminal HA tag) and pCMV4a (C-terminal FLAG tag) for mammalian expression. To create AEG-1-C75S constructs, site-directed mutagenesis via QuickChange PCR amplification was performed using the following primers: forward: 5’-GGCCGCGGCTAGCGCCGGCG-3′; reverse: 5′- TAGCCGCGGCCCAGCCGT AGCC-3′. pCMV5-FLAG-RalA plasmid was described previously.^38^

### Cell Culture

HEK293T cells were cultured in DMEM media (11965–092, Gibco) with 10% calf serum (C8056, Sigma-Aldrich). For DHHC6 shRNA stable knockdown, lentivirus was generated by cotransfection of pLKO.1 with shRNA sequences specific to DHHC6 (TRCN0000365339 or TRCN0000365405, Millipore), pCMV-dR8.2, and pMD2.G plasmids into HEK293T cells. The cell medium was collected 48 h after transfection and used to infect early passage cultures of HEK293T cells. After 72 h, infected cells were treated with 1.5 μg/mL puromycin to select for stably incorporated shRNA constructs. The empty pLKO.1 vector was used as a negative control.

### Detection of Fatty-Acylation by In-Gel Fluorescence

AEG-1 expression plasmid was transfected into HEK293T cells using PEI transfection reagent following the manufacturer’s protocol. The pCMV-Tag 4a empty vector was used as the negative control. After overnight transfection, cells were treated with 50 μM Alk14 for 6 h. The cells were washed 2× with ice cold PBS and collected by centrifugation at 1000*g* for 5 min. Cells were then lysed in 1 mL of 1% NP-40 lysis buffer (25 mM Tris–HCl, pH 7.8, 150 mM NaCl, 10% glycerol and 1% NP-40) with protease inhibitor cocktail (1:100 dilution) by rocking at 4 °C for 30 min. After centrifugation at 17,000*g* for 30 min, the supernatant was collected and protein concentration was determined with the Bradford assay (23200, Thermo Fisher). Lysates at a concentration of 0.5–2 mg/mL were incubated with 20 μL of anti-FLAG affinity gel per mg of protein at 4 °C for 2 h. The affinity gel was washed three times with washing buffer (25 mM Tris–HCl, pH 7.8, 150 mM NaCl, 0.2% NP-40). Beads were dried with gel loading tips and resuspended in 20 μL of washing buffer. TAMRA-N_3_ (47130, Lumiprobe, 1 μL of 2 mM solution in DMF), Tris[(1-benzyl-1*H*-1,2,3-triazol-4-yl)methyl]amine (TBTA) (T2993, TCI Chemicals, 1 μL of 10 mM solution in DMF), CuSO_4_ (1 μL of 40 mM solution in water), and Tris(2-carboxyethyl)phosphine (TCEP) (580560, Millipore, 1 μL of 40 mM solution in water) were added into the reaction mixture in the order listed. The click chemistry reaction was allowed to proceed at room temperature (RT) for 30 min. The reaction was quenched by adding 10 μL of 6× SDS loading dye to achieve a final concentration of ∼2× and then boiled at 95 °C for 5 min. For samples treated with hydroxylamine to remove cysteine fatty-acylation, 10 μL of the quenched reaction was treated with 2 μL of 4 M hydroxylamine (438227, Sigma, pH 7.4) and boiled at 95 °C for another 5 min. The samples were then resolved by 12% SDS-PAGE. The gel was then incubated with destaining buffer (50% CH_3_OH, 40% water and 10% acetic acid) by shaking 2–8 h at 4 °C and then incubated in water for 2 h. The gel was scanned to record the rhodamine fluorescence signal using a ChemiDoc MP (Bio-Rad). After scanning, the gel was stained with Coomassie Brilliant Blue (CBB) (B7920, Sigma) to check for protein loading.

### Acyl-Biotin Exchange

HEK293T cells were washed and collected as above. Cell pellets were suspended in 1 mL of lysis buffer (100 mM Tris–HCl pH 7.2, 150 mM NaCl, 2.5% SDS, inhibitor cocktail) with 50 mM fresh *N*-ethylmaleimide (NEM) (E3876, Sigma) in ethanol and 50 U mL^–1^ nuclease (88700, Thermo Fisher). Lysates were brought up to 1.1 mL of equal protein concentrations no greater than 2 mg/mL and allowed to block at RT for 1 h with gentle mixing. A total of 12 μL of 1,2-dimethyl-1,3-butadiene was added to lysates and allowed to react with NEM (to enhance subsequent extraction) at RT for 1 h with gentle mixing. A total of 110 μL of chloroform was added, and the mixture was shaken at RT for 5 min and then centrifuged at 17,000*g* for 1 min. A total of 500 μL of the upper aqueous phase was transferred into two separate microcentrifuge tubes with either 500 μL of 1 M hydroxylamine or 500 μL of 1 M NaCl. A total of 100 μL of 1 mM biotin-HPDP (16459, Cayman Chemical) in DMSO was added to each tube, and the samples were incubated at RT for 2 h with gentle mixing. Samples were then precipitated with cold methanol/chloroform/water (v/v 1:4:3) by centrifuging at 4,696*g* for 30 min. Samples were washed with cold methanol, briefly air-dried, and then resuspended in 200 μL of resuspension buffer (100 mM Tris–HCl pH 7.2, 2% SDS, 8 M urea, 5 mM EDTA). For each sample, 10 μL was used as loading control and 185 μL was diluted 1:10 with PBS and incubated with 20 μL of streptavidin beads with rocking overnight at 4 °C. Beads were washed 2 times with PBS containing 1% SDS and once with PBS. Beads were resuspended in 50 μL of PBS and boiled with SDS loading dye, along with input samples at 95 °C for 5 min. Samples were analyzed via western blot.

### Construction of AEG-1-C75S Mice

AEG-1-C75S mice were created in C57BL/6/J background using CRISPR/Cas9 technology.^22^ The targeting strategy is shown in Figure 4A–C. AEG-1-C75S heterozygote × heterozygote mating was performed to generate AEG-1-WT and AEG-1-C75S littermates. Mice were genotyped using an allele-specific PCR strategy (Figure 4D,E). This strategy involved two separate PCR reactions employing a common forward primer paired with either a C75S-specific reverse primer or a WT-specific reverse primer, yielding a 422 bp product size in either case (Figure 4D). An internal control primer pair (SCP2), generating a 732 bp product, was included in all reactions. The sequences of the primers are as follows: AEG-1-WT forward: 5’-AGACTGGGAACCTTTGGCTCTC-3′; AEG-1-WT reverse: 5′- TTCTTGCGGGCGCCGGCGCA-3′; AEG-1-C75S reverse: 5′- TTCTTGCGGGCGCCTGCGCT -3′; SCP2 forward: 5’-CCAGTTCTGTCCTCTGCCTTTCTC-3′; SCP2 reverse: 5’-GCACCTTGAGCATTAGGTGAGATC-3′. Mice were fed regular chow. All animal studies were approved by the Institutional Animal Care and Use Committee at Virginia Commonwealth University.

### Primary Cells Isolation and Culture Conditions

Primary mouse hepatocytes were isolated and cultured in Williams E medium containing NaHCO_3_, l-glutamine, insulin (1.5 μM), and dexamethasone (0.1 μM) as described.^23^ The cells were used immediately after isolation in house and were mycoplasma free as detected by the mycoplasma detection kit (Thermo Fisher).

### Total RNA Extraction, cDNA Preparation, and RNA Sequencing (RNA-Seq)

Total RNA was extracted from AEG-1-WT and AEG-1-C75S hepatocytes using the QIAGEN miRNAeasy Mini Kit (QIAGEN, Valencia, CA). cDNA preparation was done using the ABI cDNA synthesis kit (Applied Biosystems, Foster City, CA). The RNA-Seq library was prepared using the Illumina TruSeq stranded mRNA sample preparation kit and sequenced on the Illumina HiSeq3000 platform. RNA-Seq libraries yielded about 25–40 M reads per sample. All sequencing reads were quality controlled using FastQC v0.11.2. Illumina adapters were trimmed using TrimGalore! V.0.6.6; replicates were aligned to the reference genome (UCSC mouse genome build mm10) using STAR v.2.7.9a. Mus_musculus.GRCm38.100.gtf gene definition file was used. Differential expression analysis was performed using edgeR v3.36.0. Genes having counts per million less than 2 in all samples were excluded. Differentially expressed genes were defined using false discovery rate (FDR) cut-off value of 0.05. All bioinformatics analyses were conducted in R/Bioconductor computing environment v4.0.1. The data is available on GEO under the GSE215113 accession number (reviewer access token qbilqyiqdzsjvun).

### Western Blot and Immunofluorescence (IF) Analysis

Cell lysates and tissue extracts were prepared, and Western blotting was performed as described.^6^ The primary antibodies used were anti-AEG-1 (chicken, in-house, 1:5000) and anti-GAPDH (mouse, Santa Cruz#166545, 1:1000). Hepatocytes were cultured in collagen-1 coated 4-chamber slides and IF was performed using anti-AEG-1 antibody (chicken, in-house, 1:400) as described.^24^ Images were analyzed using a Zeiss confocal laser scanning microscope.

## Results

### AEG-1 Is *S*-Palmitoylated on Cys75

**Figure 1:**
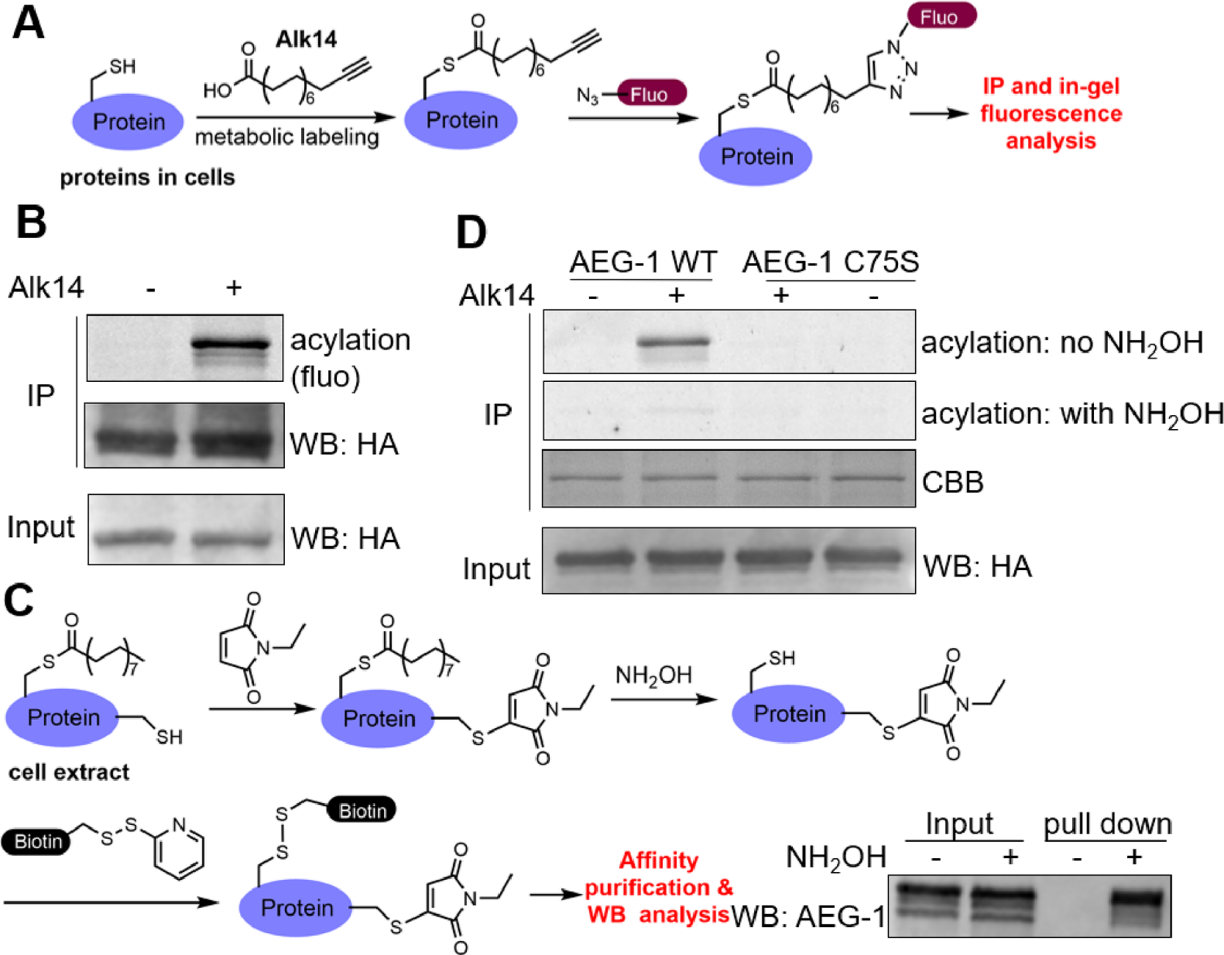
AEG-1 is palmitoylated on Cys75. (A) Scheme showing the Alk14 labeling method to detect *S*-palmitoylation. (B) AEG-1 can be labeled with Alk14 in HEK293T cells, suggesting that it has *S*-palmitoylation. (C) Scheme showing how the acyl–biotin exchange assay works. The ABE method showed that endogenous AEG-1 in HEK293T cells is *S*-palmitoylated (bottom right corner). (D) Alk14 labeling in HEK293T cells showed that AEG-1 WT is *S*-palmitoylated, but the C75S mutant is not.

We used a metabolic labeling method with an alkyne tagged palmitic acid analog, Alk14^25^ (Figure 1A), to examine whether AEG-1 is indeed *S*-palmitoylated. In HEK293T cells, we overexpressed HA-tagged AEG-1 and labeled with Alk14. After cell lysis and obtaining the protein extract, AEG-1 was isolated with HA immunoprecipitation (IP). A fluorescent tag was subsequently conjugated to the Alk14-labeled protein via click chemistry. The palmitoylation level of AEG-1 was read out by in-gel fluorescence. Indeed, we found that AEG-1 can be labeled with Alk14 (Figure 1B).

To make sure that endogenous AEG-1 is also palmitoylated, we used another well-established method called the acyl biotin exchange (ABE) assay^26,27^ (Figure 1C). In ABE, endogenous proteins in cell extract are first reacted with *N*-ethyl maleimide to block all free cysteine residues. Then, the palmitoylated cysteines are released by hydroxylamine (NH_2_OH) treatment and biotinylated using a biotin-HDPD reagent (Figure 1C). The biotinylated proteins are then affinity purified using streptavidin beads, resolved by SDS-PAGE, and blotted for the desired protein (AEG-1 in our case). The result of the ABE assay (bottom right in Figure 1C, no NH_2_OH treatment was used as a negative control) showed that endogenous AEG-1 is palmitoylated.

AEG-1 has only one Cys residue near the transmembrane helix, Cys75. To confirm that this is the palmitoylation site, we generated Cys75Ser (C75S) mutant. The C75S mutation completely abolished Alk14 labeling, supporting that Cys75 is the palmitoylation site of AEG-1 (Figure 1D). The Alk14 labeling of AEG-1 was largely removed by treatment with hydroxylamine (Figure 1D), further supporting that the palmitoylation is *S*-palmitoylation.

### AEG-1 Is Palmitoylated by an ER Palmitoyltransferase ZDHHC6

**Figure 2:**
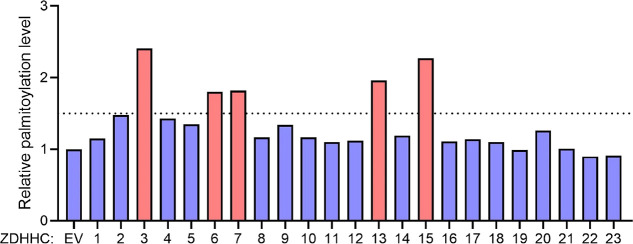
Fukata screen in HEK293T cells to identify candidate ZDHHC proteins that can palmitoylate AEG-1. The acylation level of AEG-1 was measured using Alk14 labeling and in-gel fluorescence detection. Five ZDHHC proteins (red bars) showed >1.5 fold in the AEG-1 acylation level when over-expressed.

*S*-Palmitoylation is known to be catalyzed by a family of 23 ZDHHC proteins. After establishing that AEG-1 is S-palmitoylated on Cys75, we next wanted to find out which enzyme catalyzes its palmitoylation. We overexpressed 23 mouse ZDHHC proteins in HEK293T cells (an assay called the Fukata screen^28^) and found that the expression of five of them (ZDHHC3, 6, 7, 13, 15) increased the palmitoylation level of AEG-1 by more than 1.5-fold (Figure 2).

**Figure 3:**
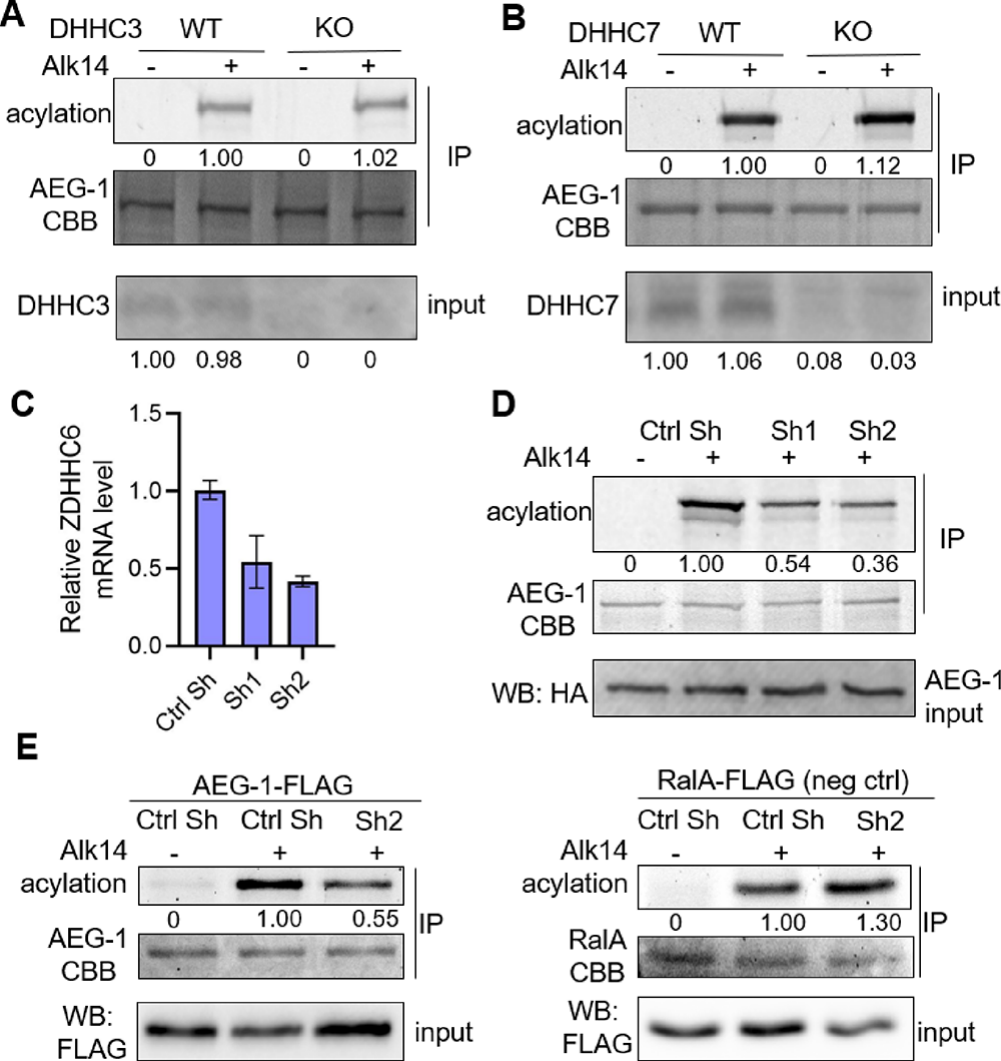
ZDHHC6 is a palmitoyltransferase for AEG-1. Knockout of ZDHHC3 (A) or ZDHHC7 (B) in HEK293T cells did not decrease AEG-1 palmitoylation as measured by Alk14 labeling (top). Knockdown of ZDHHC6 in HEK293T cells by two different shRNA (knockdown efficiency about 50% (C), decreased the palmitoylation level of AEG-1 (D). ZDHHC6 Sh2 decreased AEG-1 palmitoylation, but not the palmitoylation level of a negative control, RalA (E).

It is known that the overexpression may change the localization of the ZDHHCs and lead to modification of non-physiological substrates. Thus, typically knockdown or knockout of ZDHHCs is required to further confirm the Fukata screen. Among the five ZDHHCs that can increase AEG-1 palmitoylation, only ZDHHC6 is known to localize to the ER,^29^ where AEG-1 is localized. Thus, we hypothesized that ZDHHC6 is a physiological AEG-1 palmitoyltransferase. Indeed, the knockout of ZDHHC3 or ZDHHC7, two enzymes known to be more promiscuous, did not decrease the Alk14 labeling of AEG-1 (Figure 3A,B). In contrast, knockdown of ZDHHC6 with two different shRNA decreased the Alk14 labeling of AEG-1 (Figure 3C,D). Using ZDHHC6 sh2, we further confirmed that it can decrease AEG-1 palmitoylation but not the palmitoylation of a negative control, RalA (Ras-like protein A, Figure 3E).

### Cysteine Palmitoylation of AEG-1 Negatively Regulates Its Function

**Figure 4:**
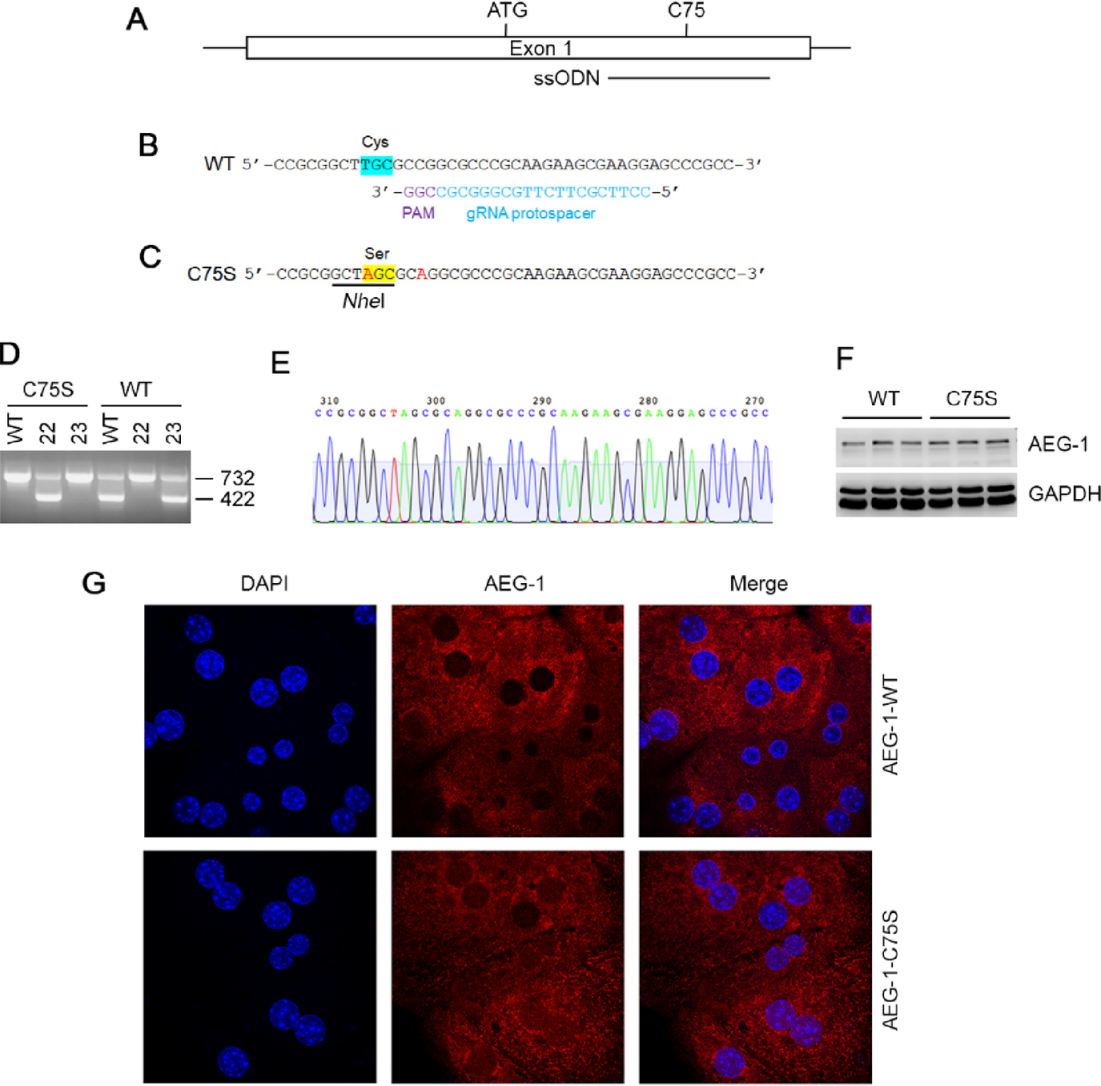
Generation of AEG-1-C75S mice. (A) Diagram of AEG-1 exon 1, indicating the locations of the ATG start codon, amino acid C75, and the 200-base sense-strand repair single stranded oligonucleotide (ssODN). (B) Partial sequence of the WT AEG-1 allele with C75 highlighted in turquoise. The sequences of the gRNA protospacer (blue) and PAM (purple) are also shown. (C) Partial sequence of the repair ssODN with C75S highlighted in yellow. The two base changes designed to create the C75S mutation and destroy the PAM sequence to prevent retargeting are shown in red. An *Nhe* I site (underlined) is also created and can be used to screen for the knock-in allele. (D) Two potential founders (22 and 23) were screened (along with a C57BL/6 WT control) using an allele-specific PCR strategy. This strategy involved two separate PCR reactions employing a common forward primer paired with either a C75S-specific reverse primer or a WT-specific reverse primer, yielding a 422-bp product in either case. An internal control (SCP2) primer pair generating a 732-bp product was included in all reactions. Lanes 1–3 correspond to the C75S-specific reactions, and lanes 4–6 correspond to the WT-specific reactions. Mouse 22 is potentially a homozygous C75S knock-in, as it is positive for the C75S allele (lane 2), and negative for the WT allele (lane 5). (E) Partial sequencing chromatograph from C75S founder 22, corresponding to the sequence of the repair ssODN in C. (F) Representative Western blot for AEG-1 in AEG-1-WT and AEG-1-C75S livers, obtained from three independent mice per group. GAPDH was used as loading control. (G) Immunofluorescence analysis of AEG-1 expression in AEG-1-WT and AEG-1-C75S primary hepatocytes. The image was analyzed by a confocal laser scanning microscope. Magnification 630×.

*S*-palmitoylation typically affects the membrane localization of proteins. However, in the case of AEG-1, we did not see the ER localization change comparing AEG-1-C75S to WT in HEK293T cells. To interrogate how cysteine palmitoylation affects AEG-1 function, we generated a genome-edited mouse strain in which Cys75 was mutated to serine (C75S) using CRISPR/Cas9 technology (Figure 4A–E). AEG-1-C75S heterozygotes were mated to generate AEG-1-WT and AEG-1-C75S littermates. Compared to AEG-1-WT, AEG-1-C75S littermates did not show any developmental anomalies, and they grew normally with comparable body weights. The frequency of AEG-1-WT and AEG-1-C75S litters showed Mendelian distribution indicating lack of embryonic lethality. The histology of internal organs did not show any anatomical or architectural abnormality. Western blot in livers detected AEG-1 expression in both AEG-1-WT and AEG-1-C75S, indicating that the mutation did not interfere with AEG-1 expression (Figure 4F). Immunofluorescence analysis of AEG-1-WT and AEG-1-C75S hepatocytes showed similar subcellular localization (Figure 4G).

**Figure 5:**
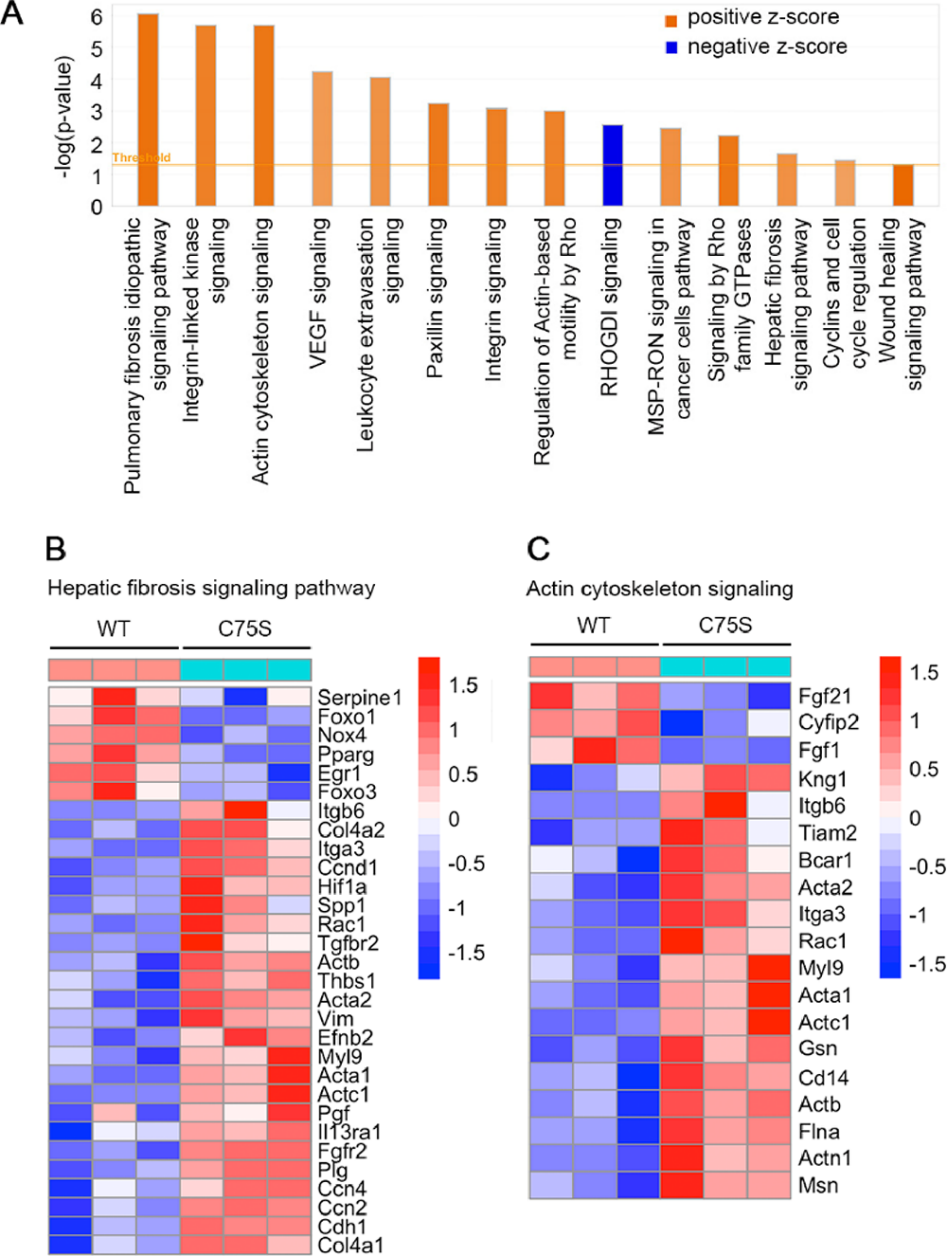
Signaling pathways activated in AEG-1-C75S hepatocytes. (A) Canonical pathways activated or inhibited in AEG-1-C75S hepatocytes compared to AEG-1-WT identified by IPA of RNA-Seq data. Signaling pathways with −log(*p*-value) of 1.3 and higher, and *z*-score of >2.0 or <2.0 are shown. VEGF: vascular endothelial growth factor; MSP-RON: macrophage-stimulating protein-recepteur d’origine nantais. (B). Heatmap of genes in the hepatic fibrosis signaling pathway. (C) Heatmap of genes in the actin cytoskeleton signaling pathway.

Although there was no morphological or histological abnormality, we checked whether AEG-1-C75S differs from AEG-1-WT at the gene expression level. We performed RNA-Seq on naïve hepatocytes isolated from AEG-1-WT and AEG-1-C75S littermates. With an FDR cut-off of 0.05 or less, 561 genes were differentially expressed, out of which 300 were upregulated, while 261 were downregulated in AEG-1-C75S hepatocytes compared to AEG-1-WT. These differentially expressed genes (DEGs) were subjected to ingenuity pathway analysis (IPA) to identify canonical pathways and upstream regulators. Figure 5A shows canonical pathways that are activated or inhibited in AEG-1-C75S compared to AEG-1-WT. A *z*-score of >2 indicates activation and a *z*-score of <−2 indicates inhibition by the C75S mutant.

These pathways could be broadly clustered into four groups: (1) pathways associated with actin cytoskeleton-regulated cell motility, such as actin cytoskeleton signaling, paxillin signaling, integrin signaling, leukocyte extravasation signaling, regulation of Actin-based motility by Rho, and signaling by Rho family GTPases; (2) pathways associated with fibrosis, such as pulmonary fibrosis idiopathic signaling pathway, hepatic fibrosis signaling pathway, and wound healing signaling pathway; (3) pathways associated with angiogenesis, such as VEGF signaling; and (4) pathways associated with cell proliferation, such as integrin-linked kinase signaling and cyclins and cell cycle regulation. All the pathways were activated, except for RHOGDI signaling, a negative regulator of Rho GTPase signaling, which showed marked inhibition. Figure 5B shows the heatmap of the genes in the hepatic fibrosis signaling pathway, which include collagens, such as Col4a1 and Col4a2, and vimentin (Vim). Figure 5C shows the heatmap of the genes in the actin cytoskeleton signaling, showing induction of several actins, such as Acta1, Acta2, Actb and Actc1, myosin (Myl9), and gelsolin (Gsn).

**Figure 6:**
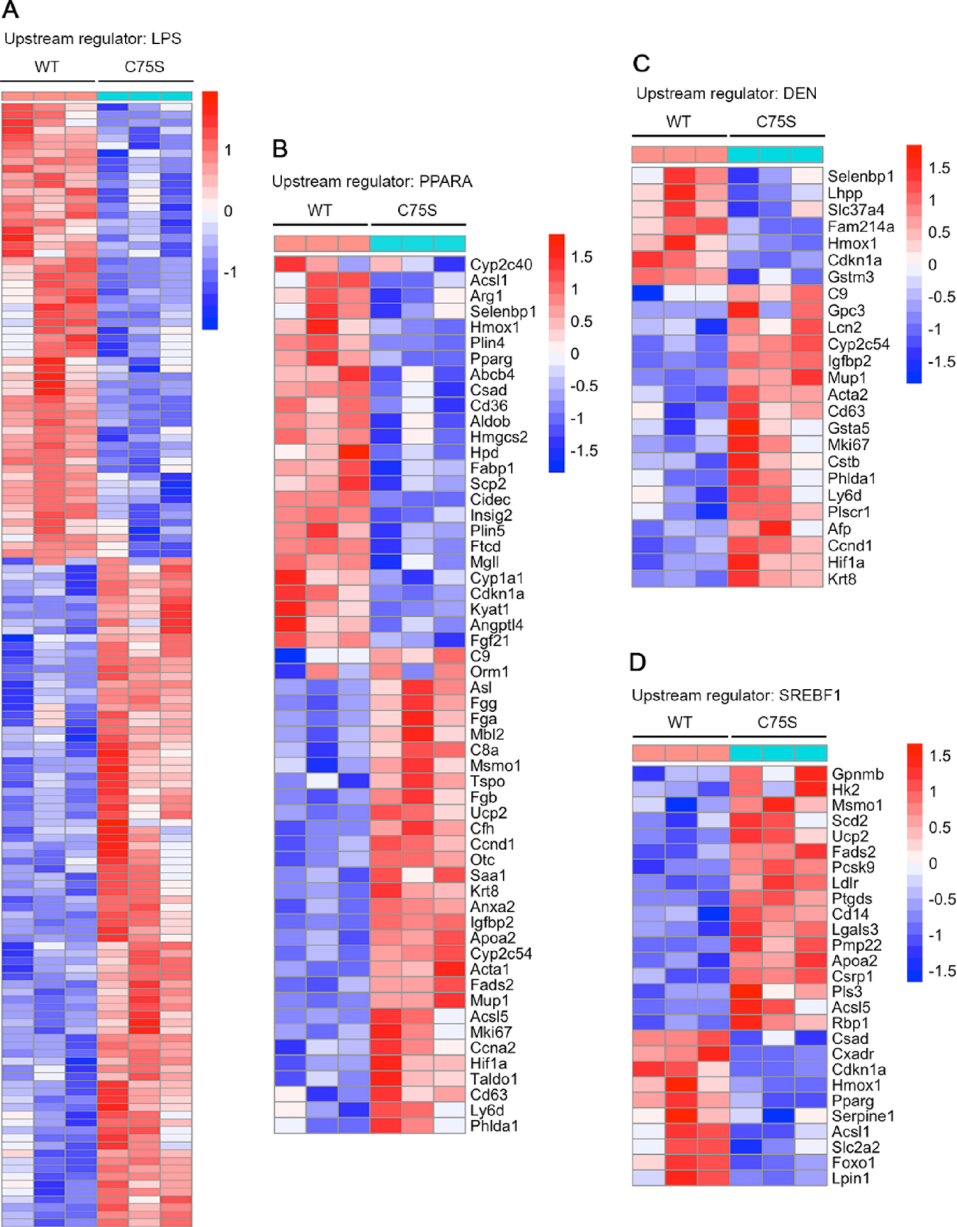
Upstream regulators of DEGs in AEG-1-C75S hepatocytes. (A) Heatmap of genes with upstream regulator lipopolysaccharide (LPS). (B) Heatmap of genes with upstream regulator peroxisome proliferator activated receptor-α (PPARA). (C) Heatmap of genes with upstream regulator diethylnitrosamine (DEN). (D) Heatmap of genes with upstream regulator sterol regulatory element binding transcription factor 1 (SREBF1).

The upstream regulators of the DEGs were checked (Table 1). The upstream regulator that showed the most significant activation in AEG-1-C75S is lipopolysaccharide (LPS), a classical inducer of inflammation. Out of the 561 DEGs, 144 genes are LPS-regulated genes, the heatmap of which is shown in Figure 6A, and the corresponding gene is listed in Table S1. The upstream regulator that showed the most significant inhibition in AEG-1-C75S is PPARα, a key regulator of fatty acid β-oxidation (Table 1), and the corresponding heatmap of the genes is shown in Figure 6B. One upstream regulator, which is significantly activated, is diethylnitrosamine, which is a potent hepatocarcinogen (Table 1). The genes included in this list include markers of HCC, such as glypican 3 (Gpc3) and α-feto protein (Afp), genes regulating cell proliferation, such as cyclin D1 (Ccnd1), p21 (Cdkn1a), and Ki-67 (Mki67), and genes regulating angiogenesis, such as Hif1a (Figure 6C). Along this line, several upstream regulators affecting cell growth and proliferation were activated in AEG-1-C75S, which include YAP1, IGF1, CTNNB1, and FOS with corresponding inhibition of CDKN1A and let-7, a miRNA that negatively regulates Ras (Table 1). Several upstream regulators affecting cell motility (including SRC and ROCK1), inflammation (including LPS, IL-6, IKBKB, and IL-2), and triglyceride and cholesterol synthesis (including SREBF1 and SREBF2), were activated in AEG-1-C75S (Table 1 and Figure 6D). IFNB1, mediating anti-tumor and anti-viral immune response, showed significant inhibition in AEG-1-C75S hepatocytes compared to AEG-1-WT (Table 1).

**Table 1:** Upstream Regulators of Differentially Expressed Genes (DEGs) in AEG-1-C75S Hepatocytes Compared to AEG-1-WT^a^

upstream regulator	*z*-score	*p*-value
LPS	3.623	2.83 × 10^–31^
IL-6	2.164	4.54 × 10^–20^
diethylnitrosamine	2.558	7.2 × 10^–19^
CEBPB	2.079	1.69 × 10^–18^
IKBKB	2.717	1.77 × 10^–15^
SREBF1	2.392	5.86 × 10^–13^
YAP1	2.948	2.86 × 10^–12^
IGF1	2.273	7.27 × 10^–11^
CTNNB1	3.196	3.23 × 10^–10^
FOS	2.591	3.82 × 10^–10^
SREBF2	2.059	1.54 × 10^–7^
SRC	2.419	2.92 × 10^–7^
IL-2	2.382	2.92 × 10^–6^
NOTCH2	2.19	3.21 × 10^–5^
ROCK1	2.411	4.54 × 10^–5^
PPARA	–2.553	1.55 × 10^–29^
CDKN1A	–2.059	1.69 × 10^–12^
corticosterone	–2.749	3.55 × 10^–11^
PPARGC1A	–2.481	2.59 × 10^–8^
let-7	–2.355	7.47 × 10^–7^
IFNB1	–2.318	2.72 × 10^–5^

a LPS: lipopolysaccharide; IL-6: interleukin-6; CEBPB: CCAAT/enhancer binding protein beta; inhibitor of nuclear factor kappa B kinase subunit beta; SREBF1: sterol regulatory element binding transcription factor 1; YAP1: Yes1 associated transcriptional regulator; IGF1: insulin-like growth factor 1; CTNNB1: catenin beta 1; FOS: Fos proto-oncogene, AP-1 transcription factor subunit; SREBF2: sterol regulatory element binding transcription factor 2; SRC: proto-oncogene c-src, nonreceptor tyrosine kinase; IL-2: interleukin-2; NOTCH2: notch receptor 2; ROCK1: rho associated coiled-coil containing protein kinase 1; PPARA: peroxisome proliferator activated receptor alpha; CDKN1A: cyclin-dependent kinase inhibitor 1A/p21; PPARGC1A: peroxisome proliferator activated receptor gamma, coactivator 1 alpha/PGC-1alpha; let-7: microRNA let-7; IFNB1: interferon beta 1. Upstream regulators of DEGs were identified by ingenuity pathway analysis (IPA). A *z*-score of >2 indicates activation, and a *z*-score of <−2 indicates inhibition by AEG-1-C75S

**Figure 7:**
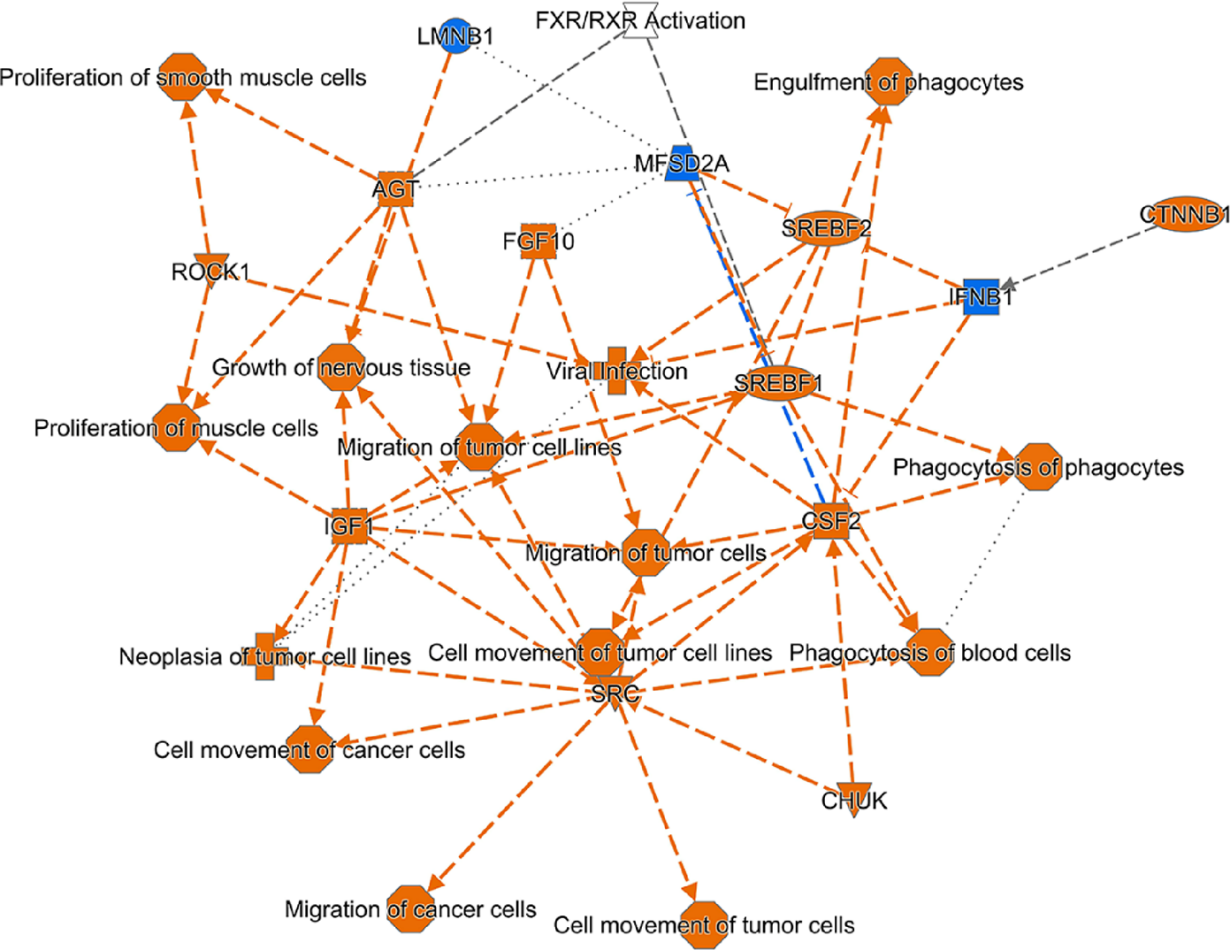
Graphical summary of cellular functions and molecules activated or inhibited in AEG-1-C75S hepatocytes. Orange color indicates an activation *z*-score of >2, while blue color indicates an inhibition *z*-score of < −2. The shapes indicate the following: octagon: function; cross: disease; inverted triangle: kinase; ellipse: transcription regulator; square: cytokine; trapezoid: transporter; circle: molecule of other class; hour glass: canonical pathway.

Figure 7 shows a graphical summary of the consequences of these gene expression changes. Cell proliferation and motility are recurrent motifs identified upon network analysis of the DEGs and their upstream regulators.

## Discussion

Our data here established that AEG-1, a key regulator of HCC and NASH, is *S*-palmitoylated on Cys75 by a palmitoyltransferase ZDHHC6 localized in the ER. Because we saw no changes in the ER localization by Cys75 *S*-palmitoylation, to investigate the effect of AEG-1 *S*-palmitoylation, we generated the CRISPR/Cas9-edited AEG-1-C75S mutant mice. We did not observe any developmental or growth anomaly in AEG-1-C75S mice compared to AEG-1-WT. This is not unanticipated because the total AEG-1 knockout mice (AEG-1–/−) did not show any such abnormality. However, AEG-1–/– mice showed a smaller litter size, compared to AEG-1+/+, which was attributed to a defect in spermatogenesis.^30^ The litter size and distribution frequency of AEG-1-WT and AEG-1-C75S mice were normal, indicating that AEG-1-C75S has a milder and different phenotype compare to AEG-1–/– mice and suggesting that C75S mutation did not nullify AEG-1 function.

Indeed, our gene expression analysis suggests a potential dominant positive effect of AEG-1-C75S. AEG-1 overexpression, such as in hepatocyte-specific AEG-1 overexpressing transgenic mice (Alb/AEG-1), causes increased proliferation, migration, invasion, angiogenesis, inflammation, and ultimately increased HCC formation. The gene expression changes we observed in AEG-1-C75S hepatocytes are consistent with Alb/AEG-1 mice. AEG-1 is a potent regulator of inflammation via its ability to activate the NF-κB pathway.^6,31^ Indeed, in our transcription analysis, the most significant upstream regulator activated in AEG-1-C75S hepatocytes, is LPS and other pro-inflammatory molecules, such as IKBKB, IL-2, and IL-6. As a corollary, the anti-inflammatory regulator corticosterone was significantly inhibited (Table 1). AEG-1 overexpression causes activation of many pro-proliferative signaling pathways, including the transcription factor AP-1.^7^ In AEG-1-C75S hepatocytes, activation of multiple pro-proliferative molecules, such as cyclin D1, Ki-67, β-catenin, YAP, IGF-1, and Fos, and inhibition of p21 and negative regulator of Ras, let-7, were observed. Activation of pro-migratory molecules, such as Src and Rock1, inhibition of type I interferon mediating anti-tumor immune response, and induction of HCC markers, such as Afp and Gpc3, indicate that AEG-1-C75S mice may harbor a pro-tumorigenic milieu, and we speculate that with age, these mice might develop spontaneous tumors or if treated with a carcinogen, such as DEN, they might develop aggressive, metastatic tumors compared to their AEG-1-WT counterparts. Long-term in vivo studies will help unravel this hypothesis and confirm these transcriptional profiling data.

Apart from its oncogenic function, AEG-1 plays a key role in regulating lipid metabolism, so that Alb/AEG-1 mice develop spontaneous NASH, while AEG-1^ΔHEP^ is protected from HFD-induced NASH.^18^ AEG-1 harbors an LXXLL motif with which it interacts with nuclear receptor retinoid x receptor (RXR).^32^ RXR is a ligand-dependent transcription factor, which, upon ligand binding, recruits transcription coactivators via LXXLL motif and stimulates transcription.^32^ Interaction of AEG-1 with RXR prevents coactivator recruitment and thereby inhibits RXR-dependent transcription.^32^ RXR serves as an obligate heterodimer partner of other ligand-dependent nuclear receptors, such as thyroid hormone receptor, retinoic acid receptor, and receptors that regulate lipid metabolism, such as PPAR, LXR, and FXR, and in in vitro assays, AEG-1 inhibits transcriptional activity of all these nuclear receptors.^8,32,33^ However, in vivo this inhibitory function of AEG-1 is skewed towards PPARα.^18^ PPARα is a master regulator of fatty acid β-oxidation. In Alb/AEG-1 mice, PPARα is inhibited, causing inhibition of fatty acid oxidation and accumulation of triglyceride.^18^ On the contrary, in AEG-1^ΔHEP^ mice, PPARα is activated, thereby precluding triglyceride accumulation. Alb/AEG-1 mice also show activation of SREBF1 and SREBF2, the former activating transcription of lipogenic genes, while the latter activating transcription of cholesterol-synthesizing genes.^18^ Similar activation of SREBF1 and SREBF2 is also seen in AEG-1-C75S hepatocytes. Collectively, it might be inferred that AEG-1-C75S hepatocytes resemble Alb/AEG-1 hepatocytes, and with age, AEG-1-C75S mice might also develop spontaneous NASH.

Overall, our findings indicate that cysteine palmitoylation might negatively regulate AEG-1 function and keep its oncogenic functions in check. For most proteins, cysteine palmitoylation is required for proper functioning. Acylation facilitates localization of Ras at the cell membrane, and inhibition of acylating enzymes is one way to check oncogenic functions of Ras.^34^ Cysteine palmitoylation is also necessary for proper functioning of STAT3.^35^ The observation that inhibition of cysteine palmitoylation might confer oncogenic activity to AEG-1 is unique, thereby unraveling a new role of acylation in protein function.

The finding that ZDHHC6 can palmitoylate AEG-1 to potentially regulate inflammation is consistent with other reports that connect ZDHHC6 to inflammation. For example, ZDHHC6 is reported to palmitoylate MYD88 and promote Toll-like receptor signaling.^36^ ZDHHC6 also palmitoylates CD36, which is reported to promote lipid uptake in macrophages and the formation of foam cells.^37^ The promotion of lipid uptake by ZDHHC6 via CD36 is particularly interesting and may also be connected to liver diseases, such as NASH.

The exact mechanism via which *S*-palmitoylation negatively affects AEG-1 function awaits further studies in the future. Currently, it is difficult to study this because although AEG-1 affects various biological pathways, its exact molecular function is still not understood except that it may interact with proteins and RNA. Analysis of protein and/or RNA interactome of AEG-1-WT and AEG-1-C75 might provide in-depth molecular insights of the mechanism via which *S*-palmitoylation negatively affects AEG-1 function.

Given that AEG-1 is a key regulator of NASH and HCC and that AEG-1 *S*-palmitoylation negatively regulates AEG-1 function and keeps its inflammatory and oncogenic functions in check, it would be worthwhile to identify the depalmitoylases that can remove the palmitoylation of AEG-1. Inhibiting the depalmitoylase would increase AEG-1 palmitoylation and thus suppress NASH and HCC.

## Conclusions

Our study demonstrates that AEG-1 is *S*-palmitoylated on Cys75 by a palmitoyltransferase ZDHHC6. Gene expression analysis indicates that *S*-palmitoylation might negatively regulate AEG-1’s oncogenic function. This identifies a previously unknown regulatory mechanism of AEG-1, which is a key regulator of HCC and NASH. This finding may therefore help to design new therapeutic strategies to treat NASH and HCC.

## Supporting Information Available

(Table S1) List of 144 genes that are differentially expressed in AEG-1-C75S and downstream of LPS (XLSX)

## Accession Codes

Human AEG1 (Q86UE4), mouse AEG1 (Q80WJ7), human ZDHHC6 (Q9H6R6), and mouse ZDHHC6 (Q9CPV7).
